# Efficient Production of Recombinant Human Brain-Derived Neurotrophic Factor in *Escherichia coli* Through the Engineering of Its Pro-Region

**DOI:** 10.3390/ijms252413425

**Published:** 2024-12-14

**Authors:** Elisa Spaccapaniccia, Tiziano Cazzorla, Daniela Rossetti, Lucio De Simone, Maria Irene Antonangeli, Andrea Antonosante, Francesca Galli, Franca Cattani, Mariano Maffei, Franck Martin

**Affiliations:** 1 Dompé Farmaceutici S.p.A., Via Campo di Pile, Nucleo Industriale Pile, 67100 L’Aquila, Italyfranca.cattani@dompe.com (F.C.); mariano.maffei@dompe.com (M.M.); 2 Altadoc S.r.l., Via Della Stazione, 24, Celano, 67043 L’Aquila, Italy

**Keywords:** neurotrophin, BDNF, recombinant, protein engineering, trypsin, *Escherichia coli*, high yield, inclusion bodies, refolding

## Abstract

Thus far, no manufacturing process able to support industrialization has been reported for the recombinant human brain-derived neurotrophic factor (rhBDNF). Here, we described the setup of a new protocol for its production in *Escherichia coli* (*E. coli*) and its purification to homogeneity. A synthetic gene, codifying for the neurotrophin precursor, was inserted into an *E. coli* expression vector and transformed into BL21 (DE3) strain. The recombinant protein accumulates, at high yields, into inclusion bodies. With the developed strategy, more than 50% of the precursor can be refolded. The protein is successively digested by trypsin and the rhBDNF mature form is finally purified by two additional chromatographic steps If the wild-type precursor can be efficiently obtained by the proposed methodology, its pro-peptide remotion, through enzymatic digestion, is however problematic. To circumvent this difficulty, the precursor hinge region, containing the natural furin recognition site, was engineered to be more specifically cleaved by trypsin. Notwithstanding the substitution of three residues in the pro-region carboxyterminal, the precursor correctly refolds and is efficiently cleaved to generate a biologically active mature rhBDNF. This efficient high-yield process fills the current need of a scalable protocol to produce GMP-grade material and unlocks the rhBDNF employment in future clinical investigations.

## 1. Introduction

Neurotrophins (NTs) are a family of closely related proteins composed of the NGF (Nerve Growth Factor), BDNF (brain-derived neurotrophic factor), NT-3 (neurotrophin-3), and NT-4/5 (neurotrophin 4-5), which are involved in survival, development, and function of neurons in the central and peripheral nervous systems [1]. NTs are synthesized as precursor pro-proteins [2] and successively subjected to proteolytic cleavage to release mature forms that elicit, as homodimers, their biological effects through the binding of one or more of the three members of the tropomyosin-related kinase (Trk) family of receptor tyrosine kinases (TrkA, TrkB, and TrkC) [3]. Furthermore, all neurotrophins, both as pro- and mature forms, can activate the p75 receptor [4], a member of the tumor necrosis factor receptor superfamily.

As neurotrophins can have broad therapeutic applications [5,6], the setup of suitable expression technologies and the development of new robust processes for these potential pharmaceutical proteins are of utmost relevance. This challenging path is always the first mandatory step toward their clinical application.

Since efforts have been made for the neurotrophin family for the successful production and commercialization of the recombinant human nerve growth factor (rhNGF) drug product Oxervate™, we decided to expand our knowledge focusing on the brain-derived neurotrophic factor (BDNF) [7]. The BDNF has a complex three-dimensional structure [8] and its production as a recombinant protein can be achieved both in eukaryotic and prokaryotic cells [9]. In eukaryotes, the protein can be produced in a soluble form, but in small quantities [10]; whereas, in prokaryotes, it can be obtained in large amounts, but insoluble [11]. Saisawang et al. recently published the production of soluble BDNF in SH-SY5Y neuroblastoma cells, reporting an overall process final yield of 250 µg of purified protein per liter of culture [4], a limited amount for process industrialization, which is a propaedeutic step to produce GMP-grade material and to perform clinical studies. When BDNF is produced in prokaryotes, as *E. coli*, its structure is too complex to be correctly folded in the microorganism cytoplasm and the recombinant protein accumulates in an insoluble form in inclusion bodies. These aggregates need to be completely denatured and refolded before starting the downstream purification process.

To achieve a BDNF recombinant expression in *E. coli*, we decided to express the human protein, together with its pro-region, as naturally occurring. The pro-region acts as a BDNF self-natural optimized chaperon and helps the mature part to refold in vivo and in vitro. This has been elegantly demonstrated by Rattenholl et al. for the NGF [12]. In the neurotrophin family, pro-domains have independently evolved over time, maintaining their chaperon activities and specificity for their adjacent mature regions. Although all four neurotrophins present around 50% of sequence similarities in their mature portions, their pro-regions highly differ in terms of amino-acid composition. In fact, Hauburger et al. have demonstrated that only the nerve growth factor pro-region can be swapped with NT3s, and vice versa, without affecting the chimeric protein refolding ability [13]. This complementarity is conserved exclusively for those two NTs.

In this work, we describe an optimized upstream and downstream processing of the human BDNF, recombinantly produced in *E. coli* and deeply characterized by means of physico-chemical methods.

Considering the high therapeutic potential of the BDNF, the obtainment of an efficient expression and purification protocol to produce high yields of the human recombinant version of this neurotrophin paves the way for its potential pre-clinical and clinical human use.

## 2. Results

### 2.1. Expression of the Wild-Type rhProBDNF in Escherichia coli (Upstream)

**Figure 1:**
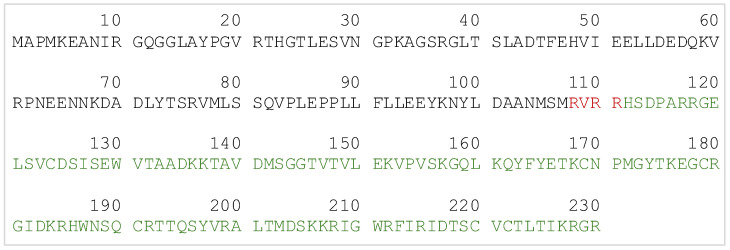
The rhProBDNF amino acid sequence as codified in the expression vector. The pro-region is reported in black, whereas the mature BDNF is reported in green. In red, the wild-type hinge region corresponding to the furin cleavage site in between the two domains. The numeration starts at the first methionine substituting the natural protein leader sequence.

A synthetic gene, based on the ProBDNF amino acid sequence, has been assembled and cloned in an expression vector downstream of the T7 promotor. The natural protein leader sequence (18 amino acids), useless for the recombinant protein expression in *E. coli*, has been substituted with a methionine to start transcription. The complete protein sequence is reported in Figure 1. The synthetic gene triplets have been specifically optimized to be translated in *E. coli*.

**Figure 2:**
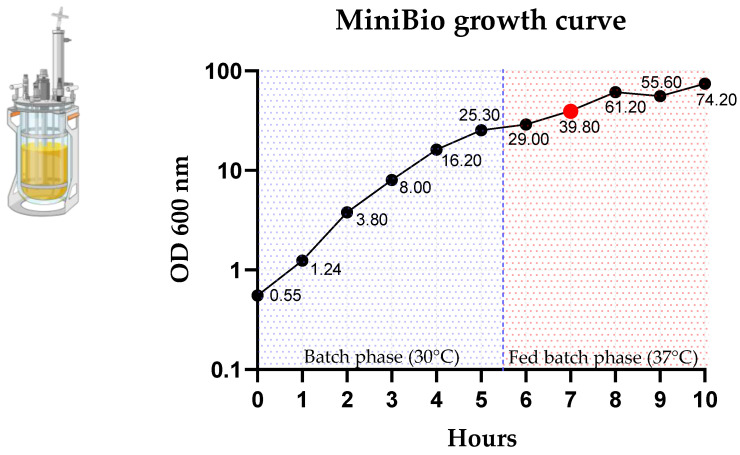
MiniBio microorganism growth curve example, measured by OD_600nm_. After 5.5 h of culture, at the end of the batch phase, temperature was shifted from 30 °C to 37 °C and the fed-batch phase started. One hour after the temperature increased, corresponding to the red dot on the curve, recombinant protein production was induced by the addition of IPTG. Fermentation was then continued for a further three hours. Fermentator icon was created with BioRender.

The expression vector has been successively transformed into *E. coli* BL21 (DE3) strain and one well isolated colony, characterized for its good recombinant protein expression has been selected to generate a research cell bank, which has been stored at −70 °C until use. One cell bank vial has been, in turn, employed to seed an overnight culture to inoculate in a fermentor the next day. For cultures, MiniBio fermenters with a 1-litre capacity (Applikon) have been used. Their capacities give very high flexibility, with short fermentation times, and the production of abundant material for downstream processing. Fermentation has been run as previously described by Bonanni et al. [14]. Briefly, fermentation has been divided in two phases. The first one at 30 °C, as batch phase, where the microorganism has its maximum growing velocity; then, once the carbon source is exhausted, a second phase at 37 °C, where growth is limited by substrate feeding. In this manner, during fermentation phases, first controlled by suboptimal temperature, then by limited substrate supply, the culture medium is always oxygenated, and cells never go into anaerobiosis, a condition needed to have an efficient production of the recombinant protein. One hour after the fed-batch had started, the recombinant protein production is triggered by isopropil-β-D-1-tiogalattopiranoside (IPTG) addition in the medium. Fermentation is then continued for a further three hours to obtain high recombinant protein accumulation in inclusion bodies, into the microorganism cytoplasm. At the end of the fermentation, biomass is harvested by centrifugation. During fermentation, key parameters, such as OD_600nm_, directly correlated to biomass and, in our case, to inclusion bodies production, are monitored. A typical fermentation growth curve is reported in Figure 2.

### 2.2. Purification of the Wild-Type rhProBDNF in Escherichia coli (Downstream)

After centrifugation, the biomass pellet was resuspended in a lysis buffer and submitted to the equivalent of four passages in a high-pressure homogenizer (GEA Niro Soavi) at 800 bars. Inclusion bodies were prepared, as described in Section 4, and denatured in guanidine. The protein solution was then refolded by dilution in a buffer containing arginine, as a solubilizing agent, ethylenediaminetetraacetic acid (EDTA), and a redox system to favor the formation of disulphide bridges. The refolding volume was calculated to dilute the guanidine present in the initial solubilized material, to a final concentration < 0.3 M. Refolding was also performed at very low protein concentration (250 µg per mL) to avoid potential aggregation phenomena and to optimize refolding yield.

After refolding, the solution was filtered to eliminate aggregates and loaded on the first chromatographic column. A hydrophobic charge-induction chromatography (HCIC) has been chosen as the capture step. This very flexible chromatography, based on the pH-dependent behavior of ionizable, dual-mode ligands, presents several advantages and, among them, allowed us to directly load the refolded protein solution on the column, without any adjustments. Due to its high hydrophobic nature, the refolded rhProBDNF efficiently binds to the column, and almost no recombinant protein is found in the column flow through. The column is then washed and re-equilibrated with a Tris buffer solution at pH 8. Elution is then achieved with a buffer containing 1 M urea and citric acid at pH 4. Those unusual conditions have been chosen to facilitate the following process phase, corresponding to the protein enzymatic digestion, which is an essential process step and removes the protein pro-peptide (see below). Citric acid has been selected for its buffering capacity between pH 4, corresponding to the HCIC elution step, and pH 6, corresponding to the enzymatic digestion. By following this, a single buffer can be used for those two successive process steps. In the same way, urea has been directly introduced in the HCIC column, to maintain the rhProBDNF protein in solution, during elution, and in the following enzymatic digestion.

Among the different HCIC resins available on the market, the MEP resin (from Pall) has been selected. This resin is composed of a cellulose matrix to which 4-mercapto-ethyl-pyridine (4-MEP) is linked, and with an average bead diameter from 80 to 100 μm, allowing high flux and low column backpressure, two well-suited characteristics for a capture step. The rhProBDNF protein binds to the resin and is eluted, under mild conditions, by dropping the pH from 8 to 4 where, acquiring a charge, the resin pyridine ring desorbs the protein.

**Figure 3:**
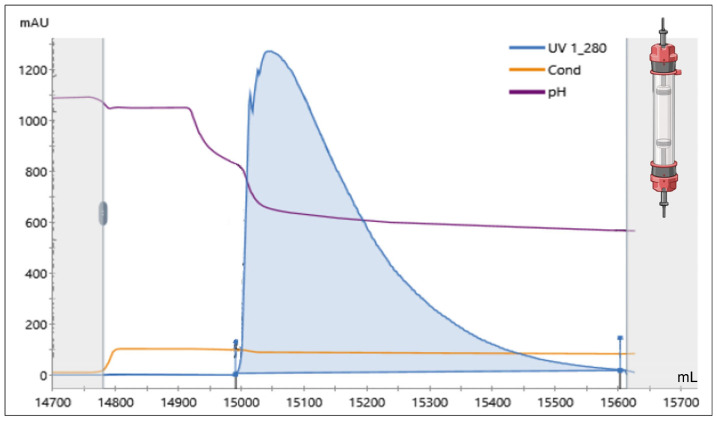
MEP column typical elution profile. After loading and washing to baseline, the protein is eluted as a single peak by dropping the pH to 4. The blue line identifies the UV detection at 280 nm, the orange line refers to the measured conductivity, while the purple line identifies the pH. Chromatography column icon was created with BioRender.

Using this approach, after refolding, the rhProBDNF protein was purified in a single chromatographic step, as determined by reversed-phase (RP) UPLC analysis, with a final purity ≥95% (Figure 3 and Table 1).

### 2.3. In Vitro Enzymatic Cleavage of the Wild-Type rhProBDNF

According to their receptor’s associations, the ProBDNF and BDNF have antagonist biological effects on cell survival. In association with p75 and sortilin, the ProBDNF induces cell death [15]; whereas, in association with p75 and/or TrkB, the BDNF supports cell survival [16]. Therefore, as we were interested in the mature form, once purified, the rhProBDNF precursor needs to be converted. In vivo, this conversion is performed intracellularly with furin [17,18] or, extracellularly, with tolloid/bone morphogenetic protein-1 (BMP-1) [19], which recognize and cleave the R^108^V^109^R^110^R^111^ sequence in the pro-region carboxy terminus. As those specific enzymes are not commercially available in GMP grade, it was important to find an alternative to set up a production process, on the correct scale. Based on the different available proteases present on the market, and on their intrinsic substrate specificities, trypsin has been chosen to digest the precursor pro-peptide, and to generate the mature BDNF form. Trypsin is a well-known protease, commercially available in different quality grades, which specifically cleaves after arginine or lysine residues [20]. As in the BDNF precursor the last pro-region residue is an arginine, trypsin is putatively compatible with the removal of the neurotrophin pro-sequence.

At this point, the MEP eluted rhProBDNF protein solution was adjusted to pH 6.00 and digested with trypsin for 16 h at room temperature, using an enzyme to protein ratio as low as 1:25,000. The RP-UPLC analysis revealed that almost 50% of the species present in the enzymatic digestion were compatible with the mature BDNF, whereas 10% of the protein corresponded to a hyperdigested variant, lacking the first six amino acids of the mature BDNF neurotrophin (ΔHSDPAR), which was further confirmed by mass spectrometry (see the Supplementary Materials). Further analyses with SDS-PAGE revealed that some hypodigested forms, resulting from incomplete pro-region digestion, were also present in the cleaved material.

**Figure 4:**
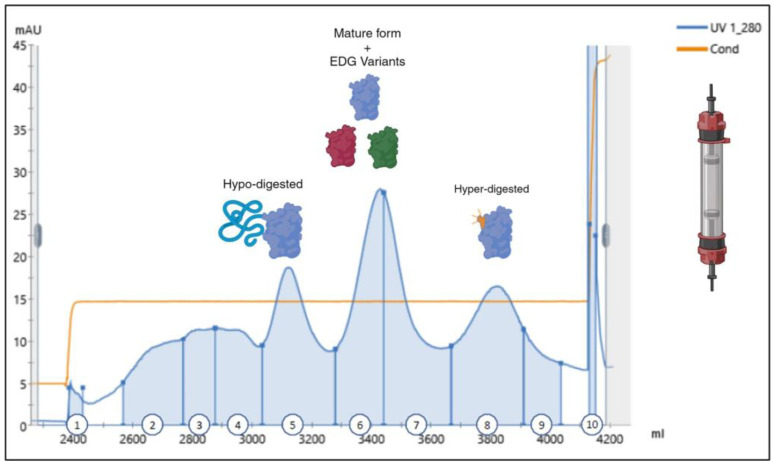
An SP Sepharose typical chromatogram of the wild-type ProBDNF digested by trypsin. The column has been eluted isocratically with a salt step. Fraction numbers are reported at the bottom of the graph. The mature BDNF protein was expected in fractions 6 and 7. Note that fraction 10, corresponding to column washing with 2 M NaCl, has a maximum absorbance of 2000 mAU. The blue line identifies the UV detection at 280 nm, the orange line refers to the measured conductivity.

As those results were in line with our expectations, enzymatic reaction was stopped by dropping the pH to 5, a value where trypsin is almost inactive, and the downstream process was continued with a cation exchange chromatography. For this, a column packed with sulphopropyl (SP) Sepharose (Cytiva) resin, pre-equilibrated at pH 5, was loaded with the digested material, washed, and finally eluted with a salt step. Elution peaks were fractionated and analyzed by the RP-UPLC. With this chromatographic approach, the digested material elutes in three different peaks: the first peak, mainly corresponding to the elution of the hypodigested material; the central portion, containing the mature BDNF; the last peak, corresponding to the hyperdigested material (Figure 4).

Of note, in this chromatography we observed that, in the central portion, which is supposed to contain the mature BDNF, the protein of interest was present, but only in marginal quantities and heavily contaminated by the hyperdigested variant, that represented up to 50% of the eluted material. We also observed an important peak in the column wash with 2 M NaCl (fraction 10), corresponding to a high percentage of the loaded material (Table 2). Mass spectrometry analysis revealed that the material present in this last chromatographic peak mainly contains two enzymatic digestion variants (EDG) with a similar fractional abundance, and corresponding to R^111^-BDNF (27%, namely EDG-V1) and V^109^R^110^R^111^-BDNF (26%, namely EDG-V2). Only a small percentage of the correctly digested mature BDNF (9%) and the hyperdigested variant (13%, ΔHSDPAR), together with other less abundant forms (oxidized, deaminated, modified, or other minor hypodigested variants) were detected by mass spectrometry (see the Supplementary Materials).

Since protein concentration of the correctly digested BDNF form was always inferior to the other contaminant species, and due to its homodimeric nature, the recombinant BDNF protein could not be purified to homogeneity, as in its dimeric form it is always associated with a hypo or a hyper-digested variant (see Section 3).

### 2.4. BDNF-Pro-Peptide Engineering to Optimize the Enzymatic Cleavage

**Figure 5:**
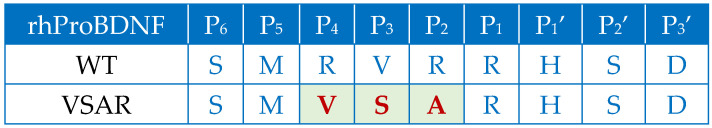
Hinge region amino-acidic sequences of the rhProBDNF wild-type cleavage site (Furin substrate) and “VSAR” variant (highlighted in red).

At this point, we decided to replace the natural R^108^V^109^R^110^R^111^ hinge region amino acid sequence with V^108^S^109^A^110^R^111^ (ProBDNF–VSAR), consequently eliminating two of three potential trypsin cleavage sites (Figure 5).

**Figure 6:**
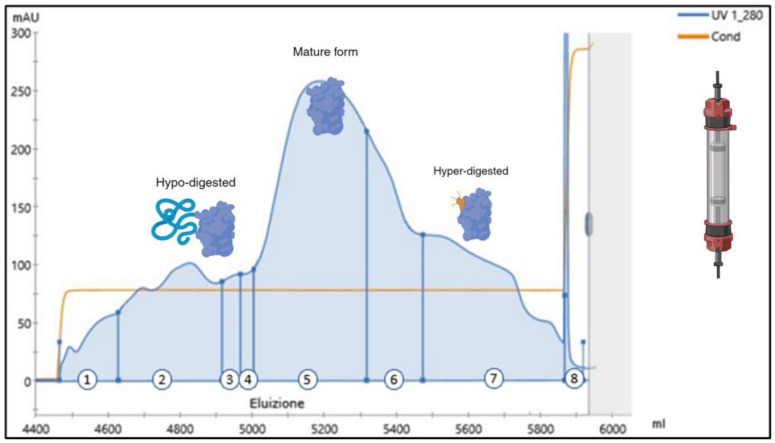
A typical SP Sepharose elution profile chromatogram of the rhBDNF–VSAR variant after trypsin digestion. Fraction numbers are reported at the bottom of the graph. The blue line monitors the UV detection at 280 nm, the orange line refers to the measured conductivity.

**Figure 7:**
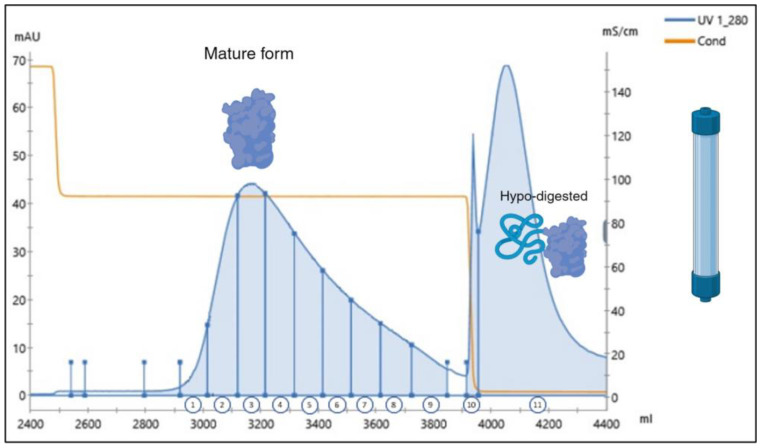
A representative phenyl Sepharose elution profile. After salt adjustment, the protein has been loaded on the HIC column and eluted with a first salt step. Fraction numbers are reported at the bottom of the graph. After elution, the column is washed by dropping salt concentration to zero. The blue line identifies the UV detection at 280 nm, the orange line refers to the measured conductivity.

To achieve this, a new synthetic gene codifying for the ProBDNF–VSAR protein was cloned into the same expression vector. Following the same protocol as described for the WT form, the rhProBDNF–VSAR protein was produced, refolded, and purified by HCIC. The rhProBDNF–VSAR was then digested by trypsin and separated on the CEX column. As can be observed in Figure 6, the obtained chromatogram importantly differed from the WT one, with the main peak (fraction 5) corresponding to the mature BDNF. This time, the RP-UPLC analysis revealed only traces of the hyperdigested variant (ΔHSDPAR), with a percentage ≤0.9% (Table 3). The selected fraction was then adjusted with salt and loaded on a phenyl Sepharose column hydrophobic interaction chromatography (HIC) to eventually separate the protein variants according to their hydrophobicities (Figure 7). The main peak was fractionated and analyzed by the RP-UPLC. At this final step, the recovered rhBDNF protein showed a purity ≥98%, and with less than 1% of hyperdigested form contaminant (Table 4).

### 2.5. Recombinant Human BDNF Protein Characterization

**Figure 8:**
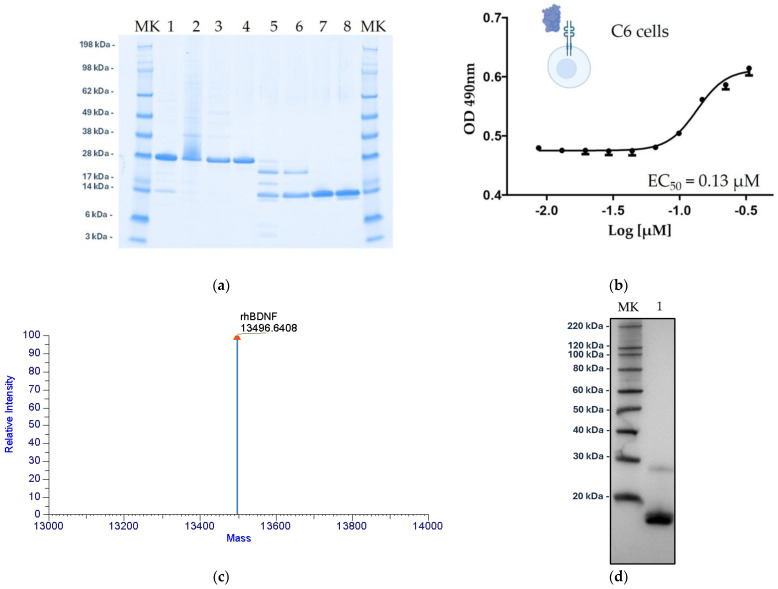
Mature rhBDNF protein characterization. (**a**) SDS-PAGE showing all the downstream process phases for rhBDNF production. Lane 1: rhProBDNF standard; Lane 2: Solubilized Inclusion Bodies; Lane 3: Refolded rhProBDNF VSAR; Lane 4: MEP fraction 1; Lane 5: Enzymatic digestion (SP T_0_ load); Lane 6: HIC Phenyl T_0_ load; Lane 7: rhBDNF API Bulk; Lane 8: rhBDNF standard (MK = molecular weight marker). SDS-PAGE was performed on a 4–12% separating gel under reducing conditions; (**b**) Dose–response curve for the rhBDNF (C6 proliferation bioassay). C6 cells were treated with the serially diluted and purified rhBDNF and 48 h later rhBDNF biological activity and EC_50_ were determined. The results shown are the average of four independent replicate plate experiment and error bars to indicate SD. (**c**) Deconvoluted spectrum of liquid chromatography–mass spectrometry (LC–MS) analysis of rhBDNF under denaturing conditions (intact molecular weight analysis, IMW). (**d**) Western blot analysis of the rhBDNF purified protein. Lane 1: rhBDNF API bulk (MK = molecular weight marker).

The purified recombinant human mature BDNF was finally characterized by means of chemico-physical, biochemical, and cell biology techniques. To confirm protein identity, purity, and its molecular characterization, SDS-PAGE, western blotting, reversed-phase UPLC, and mass-spectrometry analyses were performed on a rhBDNF sample buffer exchanged in sodium phosphate 50 mM, sodium chloride 100 mM at pH 7.0. As shown in Figure 8a, the rhBDNF migrated as a single band of approximately 13 kDa, on a 4–12% SDS-PAGE gel under reducing conditions (average theoretical mass, 13,511 Da), exhibiting a high level of purity (>95%). Moreover, the western blot analysis indicated that the protein was efficiently recognized by an anti-BDNF human antibody (Figure 8d). The experimental mass was determined by intact molecular weight analysis through LC–MS and the result was 13,496 Da (monoisotopic mass), in perfect agreement with the theoretical one (Figure 8c). The rhBDNF biological activity was finally evaluated by a proliferation assay using C6 rat glial cells expressing the BDNF natural receptor TrkB. The rhBDNF exhibited a typical dose–response curve confirming its functionality and its ability to induce proliferation of Trk-B expressing cells. The rhBDNF half maximal effective concentration (EC_50_) was determined, as reported in Figure 8b. In addition to this preliminary assay, a more detailed functional in vitro and in vivo characterization to investigate the therapeutical potential of the produced rhBDNF purified protein was performed by Pisani et al. [21].

## 3. Discussion

### 3.1. Expression and Refolding of rhProBDNF

Although refolding a protein is always challenging, recombinant protein production in inclusion bodies presents several advantages. Among them, this method often gives high production yields and, starting from gram(s) of material, eases the downstream processing. Inclusion bodies are very stable protein aggregates that can be stored for years at low temperatures before being processed, thus providing a very high flexibility in upstream and downstream scheduling. Furthermore, the starting material is weakly contaminated with DNA and lipopolysaccharide, two components which need to be efficiently removed in a protein preparation for therapeutic use. The last advantage of this strategy is that *Escherichia coli* has low production costs and, compared to eukaryotes, a reduced fermentation time, with hundreds of different strains and expression vectors commercially available.

In the BDNF case, expressing the protein together with its pro-region, facilitates its refolding. Effectively, the overall refolding yield, superior to 50%, can be considered as more than adequate for setting up an industrial process. As for the NGF, refolding the BDNF without its pro-region results in very low process yields and, consequently, in a material difficult to purify to homogeneity.

### 3.2. Trypsin Digestion in Presence of 1 M Urea and Citric Acid

After MEP column elution, the next process step to remove the pro-region was the enzymatic digestion of rhProBDNF. For this, trypsin was used since it is, to the best of our knowledge, the unique suitable enzyme commercially available in GMP grade, compatible with this process. To adjust the MEP fraction pH to 6, a value compatible with trypsin activity, and to avoid a tangential flow filtration to exchange the reaction buffer, citric acid has been already introduced in the MEP chromatography. By exploiting its different pKa, the solution pH can easily be adjusted by a simple addition of a sufficient amount of sodium hydroxide.

In a previous performed experiment, we observed that the rhProBDNF tryptic digestion releases a series of small hydrophobic peptides, which aggregate and pull down the generated rhBDNF, keeping only a small fraction of it in solution. To avoid this massive precipitation, 1 M urea was identified as the best additive to prevent this phenomenon. Whereas, in the same conditions, glycerol, trehalose, polyethylene glycol, and non-ionic detergents, tested at several concentrations, have not succeeded in preventing this precipitation.

In this process, the employment of the urea has several advantages. Even at this relatively low concentration (1 M), it helps to maintain the small hydrophobic peptides in solution, thus allowing their elimination directly in the following column flow-through. In addition, urea neither interferes with trypsin’s enzymatic activity, nor with the execution of the next process step, which in our case is a cation exchange chromatography.

For those reasons, urea has been introduced during the enzymatic digestion and, even previously, into the MEP column elution buffer to obtain a “ready to cut” material. A further advantage of this approach is that urea not only helps to maintain the rhProBDNF in solution, but it also favors its desorption from the MEP resin, at pH 4. In the presence of urea, the rhProBDNF is harvested in a sharp peak; whereas, in its absence, the rhProBDNF interacts more strongly with the resin, resulting in much higher elution volumes.

The last advantages of urea are its low cost and its efficient elimination during the following chromatography (ion exchange).

During digestion, the enzyme to protein ratio has been kept as low as 1 to 25,000. In those conditions, digestion of the rhProBDNF is completed approximately in 16 h, introducing a sufficient lap of time to perform in process controls to follow reaction evolution and completion.

### 3.3. Hypo and Hyperdigested Forms

**Figure 9:**
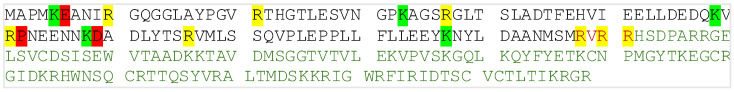
The rhProBDNF amino acid sequence and trypsin cleavage sites. Lysine (K) residues are highlighted in green, while arginines (R) in yellow. Red-highlighted residues are P_1_’ amino acids impeding trypsin cleavage. Black sequence refers to the Pro-peptide region, while green sequence to mature BDNF.

Of note in the above-described conditions, trypsin also cleaves the rhBDNF precursor pro-region more than once. By contrast, apart from the hyperdigested variant cutting site (ΔHSDPAR), the mature rhBDNF region is not further over-digested by trypsin. This may be due to the tight BDNF folding in which lysine and arginine residues, present in the mature protein, are not sterically accessible to trypsin; whereas, being less structured, the pro-region is much more exposed and therefore sensitive to the proteolytic enzyme. The pro-region possesses five lysines and eight arginines, which are putative trypsin cleavage sites (10 sites out of 13); while, due to their P1’ residue nature sequences (KE, RP, and KD), three of those sites are not cleavable (Figure 9). Therefore, during trypsin digestion, this elevated number of potential cleavage sites generates several hypodigested variants.

In our case, trypsin hydrolysis is always a compromise between pushing the digestion to completion reducing the hypodigested forms concentration, without transforming all the generated rhBDNF in the hyperdigested variant (ΔHSDPAR). Therefore, it is subsequently necessary to obtain reproducible material, containing only a limited number of species.

The situation is even more complicated by the BDNF homodimeric nature, as each mature BDNF monomer can be associated with different hypo or hyperdigested forms, and vice versa. It is consequently important to monitor the digestion kinetics to stop enzymatic reaction when the best balance has been reached. In our case, we decided to stop trypsin reaction when 80% of the mature BDNF, and no more than 15% of the hyperdigested forms, were generated.

### 3.4. Introduction of the VSAR Sequence

As it was important to develop an alternative process to furin cleavage, the substitution of the naturally occurring R^108^V^109^R^110^R^111^ sequence, with the VSAR one, has permitted to reduce the generation of enzymatic digestion variants (R^111^-BDNF and V^109^R^110^R^111^-BDNF) by trypsin, and to generate the mature rhBDNF form of interest. This enzyme, manufactured in GMP, and at a reasonable cost, is compatible with the regulatory authorities’ requests, and therefore with process industrialization. We observed that trypsin has a marked preference for the first R^108^, and the third R^110^ residues, located in the hinge region of the wild-type protein. It also seems that, once generated, those V^109^R^110^R^111^-BDNF (EDG-V2) and R^111^-BDNF (EDG-V1) variants are poor substrates for the enzyme, which do not proceed with digestion to remove those “extra” amino acids.

Unexpectedly, after the introduction of the “VSAR sequence”, the arginine number 111 (R^111^), which was not efficiently cleaved in the WT protein, becomes the main trypsin cleavage site in the rhProBDNF–VSAR mutein. As can be seen by SDS-PAGE (Figure 8, Lane 5), trypsin digestion of the rhProBDNF–VSAR mutein still generates two major hypodigested forms (higher molecular weight), but in lower amounts with respect to the produced mature rhBDNF. But, due to their significant sequence differences, those hypodigested variants can efficiently be separated by the two successive chromatographic columns, following the enzymatic digestion step.

### 3.5. High Yield Process

A further advantage of the VSAR variant is that the three amino acid substitutions, corresponding to the removal of two basic amino acids, do not interfere with refolding efficiency, which remains high and comparable to the WT one. This latter property allows us to recover elevated quantities of the rhProBDNF precursor and enables a high process yield. From the different productions performed at R&D scale, we can estimate that the overall process yield is in the range of 100 mg of the purified mature rhBDNF, per fermentation liter, which is, to the best of our knowledge, the highest ever reported yield for the recombinant human BDNF purification from an *E. coli* system production.

## 4. Materials and Methods

### 4.1. Plasmid

The recombinant protein gene, corresponding to the rhProBDNF, was cloned under the control of the T7 promotor, in a kanamycin selectable expression vector (pET28 b+, Novagen, Merck KGaA, Darmstadt, Germany, code#69865-3). The synthetic gene triplets have been optimized for expression in *Escherichia coli*.

### 4.2. Transformation, Clone Selection and Cleaned Cell Bank

The expression vector was transformed into *Escherichia coli* BL21 (DE3) strain (New England Biolabs, Ipswich, Massachusetts, MA, USA, code#C2527) following the supplier’s instructions. Once transformed, cells were plated on LB agar (Merck, Merck KGaA, Darmstadt, Germany, code #1.10283) with kanamycin (Sigma, Merck KGaA, Darmstadt, Germany, code #K1637). Four well isolated colonies were picked from cultured plates and assessed in liquid cultures, performed in a TB medium supplemented with kanamycin (Sigma, Merck KGaA, Darmstadt, Germany, code #K1637, 50 µg/mL). The best rhProBDNF expressing clone was selected to prepare the research cell bank.

### 4.3. Biomass Homogenization and Washes

At the end of fermentation, the biomass was harvested by centrifugation and the pellet resuspended in a lysis buffer composed of 0.2 M trometamol (Merck, Merck KGaA, Darmstadt, Germany, code #1.08386), 1 mM triplex (Merck, Merck KGaA, Darmstadt, Germany, code #108421), at pH 7.0. The biomass to lysis buffer ratio was 1 mL of Tris buffer for 0.2 g of cellular pellet. Resuspended biomass was homogenized at 800 bars for four passages (GEA Niro Soavi, Parma, Italy, Model Panda type NS1001L2K). After centrifugation, inclusion bodies were washed with a ratio of 1 mL of homogenized material to 0.5 mL Brij buffer 6% (Brij 35, Merck KGaA, Darmstadt, Germany, code # 801962). Two further washes were performed with Tris buffer (0.1 M Trometamol, 0.02 M Triplex, pH 7.0).

### 4.4. Solubilization and Refolding

Inclusion bodies were resuspended in 150 mL of Tris buffer and solubilized with 1.4 volume of 7.2 M guanidine hydrochloride (NIGU, Waldkraiburg, Bayern, Germany, code #140000323), 100 mM trizma base (Merck, Merck KGaA, Darmstadt, Germany, code #1.08386), 10 mM EDTA (Merck, Merck KGaA, Darmstadt, Germany, code #1.08421), 9 mM L-cysteine (Merck, Merck KGaA, Darmstadt, Germany, code #1.02735), pH 8.0. Following solubilization, refolding was performed by diluting the denatured sample into a buffer containing a red/ox system (5 mM L-cysteine, Merck, Merck KGaA, Darmstadt, Germany, code #1.02735; 1 mM L-cystine Merck, code #1.02735, Sigma, Merck KGaA, Darmstadt, Germany, code #C7602), 0.75 M arginine as solubilization agent (SHANGHAI KYOWA AMINO ACID Co., Ltd. (Shanghai, China), code #LARH25BIF13), at pH 9.1. Refolding was performed at 4 °C for 23 h.

### 4.5. HCIC Column

Refolded material was loaded onto a hydrophobic charge-induction chromatography (MEP HyperCel PALL, Port Washington, NY, USA, code# 12035-040) at pH 9.1. The column was washed with arginine 0.75 M at pH 9.1, then equilibrated with 10 mM trometamol (Merck, Merck KGaA, Darmstadt, Germany, code #1.08386) and eluted in a single fraction with 1 M urea (Merck, Merck KGaA, Darmstadt, Germany, code #15604), 50 mM citric acid (Merck, Merck KGaA, Darmstadt, Germany, code #1.00243) pH 4.0 until the signal returned to baseline.

### 4.6. Enzymatic Digestion

Enzymatic digestion was directly performed for 16 h in the HCIC elution buffer by adjusting its pH to 6.0. Trypsin (Roche, Basel, Switzerland, code #06369880103) was added at room temperature, with a ratio of 1:25,000. After the tryptic digestion was completed, the pH was adjusted to 5.0 by addition of a 1 M arginine solution in 50 mM citric acid and 1 M urea at a ratio 1/8 to inhibit trypsin activity.

### 4.7. SP-HP Column (CEX)

The column containing SP-HP (high performance) resin (Cytiva, Marlborough, MA, USA, code #71-1087-03) was equilibrated with 50 mM citric acid, 1 M urea, and 125 mM arginine at pH 5.0. After loading, the column was first washed with 1 M urea and 50 mM citric acid pH 6.5, and the protein successively isocratically eluted in 25 mM phosphate buffer pH 6.5 with a higher salt concentration. Purified fractions were analyzed by RP-UPLC analysis before pooling.

### 4.8. Phenyl Sepharose Column

The SP-HP pool was adjusted to 2 M NaCl and loaded on a phenyl Sepharose fast flow low substitution resin (Cytiva, Marlborough, MA, USA, code #71-1065-03) pre-equilibrated with 25 mM phosphate buffer, 2 M NaCl at pH 6.5. After washing, the protein was eluted by dropping salt concentration to 1 M NaCl. Purified fractions were analyzed by RP-UPLC before pooling.

### 4.9. Tangential Flow Filtration

A 5 kDa Omega cassette (Pall, Port Washington, NY, USA, code #4503685434) was used to concentrate the pooled fractions to the desired concentration. The solution was diafiltrated with 6 volumes of 50 mM phosphate buffer, 100 mM NaCl at pH 7.0. The final protein concentration was determined by UV280 nm.

### 4.10. Reverse-Phase UPLC

RP-UPLC analyses were performed on a Waters Acquity UPLC H class liquid chromatograph equipped with a PDA eλ detector (Waters). The liquid chromatography separation of the protein samples and respective impurities was performed on a C_4_ column (2.1 × 100 mm, particle size of 1.7 µm) using a gradient elution at a constant flow rate of 0.3 mL/min. Mobile phase A was a mixture of 0.1% (*v*/*v*) trifluoracetic acid (Sigma, Merck KGaA, Darmstadt, Germany, code #302031) and MilliQ water (Burlington, MA, USA), and mobile phase B was a mixture of 0.1% (*v*/*v*) trifluoracetic acid and acetonitrile (Sigma Aldrich, Merck KGaA, Darmstadt, Germany, code # 34967). Detection was carried out at 220 nm.

### 4.11. LC–MS Analysis

LC–MS analyses were carried out on a Orbitrap Fusion™ Tribrid Mass Spectrometer (ThermoFisher, Waltham, MA, USA) coupled with a Vanquish UHPLC System (ThermoFisher, Waltham, MA, USA). The determination of the molecular weight was achieved by a H-ESI mass spectrometry analysis in positive linear mode. Protein samples were separated on a C_4_ column (2.1 × 100 mm, particle size of 1.7 µm) using a gradient elution (see the RP-UPLC phases). The flow rate was maintained at 0.3 mL/min and the mass spectra were acquired in full scan mode (700–3500 Da). Data acquisition and instrument control were performed using the ThermoFisher Scientific Xcalibur 4.3 integrated software. Data analysis (protein deconvolution) were performed using ThermoFisher Scientific Bio Pharma Finder 4.0 integrated software.

### 4.12. SDS-PAGE

SDS-PAGE analysis was performed using a 4–12% Bis-Tris polyacrylamide gel (NuPage/Thermo Fisher Scientific). Electrophoresis was carried out in an Xcell SureLock 1000 with 2-(N-morpholino) ethanesulfonic acid buffer (MES; NuPage/Thermo Fisher Scientific, Massachusetts, MA, USA) at pH 7.3 containing sodium dodecyl sulfate (SDS). The protein samples were prepared in reduced conditions using LDS Sample Buffer 4× (Coomassie G250 and phenol red as tracking dyes, NuPage/Thermo Fisher Scientific Massachusetts, MA, USA, and NuPAGE Sample Reducing Agent 10× containing 500 mM dithiothreitol (DTT), NuPage/Thermo Fisher Scientific, Massachusetts, MA, USA). The mixture was vortexed and then boiled at 70 °C for 5 min. Denatured samples were loaded onto the gel together with standards of known molecular mass (SeeBlue™ Plus2 Pre-stained Protein Standard, ThermoFisher Scientific). Gels were run at a constant voltage of 200 V for 30 min and successively stained with the Colloidal Blue Staining Kit (ThermoFisher Scientific, Massachusetts, MA, USA). After staining, gels were digitalized using GS 900 (BioRad, Hercules, CA, USA), and pherograms were obtained using ImageLab 6.0.1 software (BioRad, Hercules, CA, USA)).

### 4.13. Western Blot Analysis

For the western blot analysis, about 1 µg of denatured protein was run on 4–12% Bis-Tris polyacrylamide gel (NuPage/Invitrogen code NP0002). The sample preparation and electrophoresis were carried out as described in the SDS-PAGE paragraph. Gels were electroblotted onto nitrocellulose membranes (iBlot Gel Transfer Stacks Nitrocellulose Mini, Invitrogen, Massachusetts, MA, USA, code # IB301002) using the iBlot Dry Blotting System from Invitrogen. The membranes were washed one time with PBS-T 0.1% (PBS tablets were purchased from Medicago, Quebec City, QC, Canada, code # 098912100, while Tween 20 is from Sigma Aldrich, code # P9416) then were blocked in 5% non-fat dry milk (skim milk powder, Millipore, Burlington, MA, USA, code # 70166) in PBS-T 0.1% for 1 h a RT. Membranes were then incubated with a primary human anti-BDNF polyclonal antibody (Proteintech, Rosemont, IL, USA, code # 25699-1-AP) diluted (1:1000) in 5% non-fat dry milk in PBS-T 0.1% overnight at 4 °C. After washes with PBS-T 0.1%, membranes were incubated for 1 h a RT at slow agitation with Anti-Rabbit IgG HRP-linked antibody (from Vector, Newark, NJ, USA, code # PI-1000) diluted (1:500) in 5% non-fat dry milk in PBS-T 0.1%. Magic Mark XP Western Standard (from Invitrogen, Massachusetts, MA, USA, code # LC5602) were used as molecular weight markers. Immunoreactive protein bands were detected by Super Signal West Pico Plus Chemiluminescent Substrate (purchased from Thermo Scientific, Massachusetts, MA, USA, code # 34580) and the images were acquired by using the GeneGnome system from Syngene (Genesys software version 1.7.2).

### 4.14. C6 Proliferation Assay

Rat glioma C6 cells were purchased from ATCC (catalogue # CCL-107) and cultured in DMEM medium (Gibco, Texas, TX, USA, code # 11960-044) supplemented with 10% fetal bovine serum (Gibco, Texas, TX, USA, code # A5256701), 1% penicillin–streptomycin solution stabilized 10000 U/10 mg/mL (Sigma, code # P4333) and 1% L-glutamine 200 mM (Gibco, Texas, TX, USA, catalogue # 25030-081) in a humidified atmosphere of 95% air and 5 ± 2% CO_2_ at 37 ± 2 °C. C6 cells at a confluency of 70% were detached using trypsin-EDTA (Sigma, code # T4049), harvested and centrifuged at 700 rpm for 7.5 min, then washed three times. Cells were finally re-suspended in a DMEM medium supplemented with 1% penicillin–streptomycin solution stabilized 10,000 U/10 mg/mL, 1% L-glutamine 200 mM, and 3.4 mg/mL bovine serum albumin, and seeded in 96-well plates at 5 × 10^3^ cells/wells. Proliferation effect (dose–response) of the rhBDNF at a concentration ranging from 0.23 to 9 µg/mL was tested in triplicate on a total of four 96-well plates. Microplates were then incubated for 48 ± 1 h at 37 ± 2 °C in a humidified incubator containing 95% air and 5 ± 2% CO_2_ to allow C6 cells to adhere and proliferate in response to the rhBDNF. After 48 ± 1 h-incubation, CellTiter 96^®^ AQueous One Solution Reagent (Promega, Madison, WI, USA, code # G3581) was added to the microplate wells and incubated for 4 h ± 10 min. The intensity of the colored soluble formazan produced from the cellular reduction of the MTS tetrazolium compound and directly proportional to the number of viable cells, was detected by recording the absorbance at 490 nm with a plate reader. Log rhBDNF concentration (µM) was plotted vs. mean OD_490nm_ and a 1/Y weighted four-parameter (4-PL) curve fitted. The rhBDNF EC_50_ value was calculated, for each plate, from the non-linear regression 4-PL curve.

### 4.15. UV-VIS

Total protein concentration in solution was determined by measuring ultraviolet (UV) absorption at 280 nm using UV transparent disposable micro cuvettes (BRAND^®^ UV cuvette micro, Merck, Merck KGaA, Darmstadt, Germany, code # BR759220-100EA) and a UV-VIS Spectrophotometer (Perkin Elmer, Model Lambda 35+, Waltham, MA, USA). Protein concentration was calculated according to the Lambert–Beer law by using the rhBDNF molar extinction coefficient.

## 5. Conclusions

The substitution of the R^108^V^109^R^110^R^111^ hinge region amino acid sequence with VSAR has changed the rhProBDNF mutein digestion profile, without altering protein folding, leading to the accumulation of mature rhBDNF as a main reaction product. This outstanding result has permitted us to use trypsin to generate the rhBDNF protein with high efficiency and quality. The introduction of citric acid and urea in the first column chromatography and, subsequently, in the trypsin digestion step, have allowed us to design a simple, reproducible, and robust process, which eases its scaling up in GMP plants.

The presented results, including the high process yield, allow for process industrialization to generate clinical grade material, and to fulfil the current request of new therapeutic molecules, to be tested, in not yet treatable diseases in which the BDNF plays a crucial role.

## 6. Patents

Patent application WO2024165725 [22].

## Figures and Tables

**Table 1:** RP-UPLC analysis of rhProBDNF in refolding step and HCIC fraction 1.

Samples	rhProBDNF Purity %
Refolding	89.6
Fraction 1 MEP	99.1

**Table 2:** Representative rhBDNF analysis results after SP elution step. The presence of rhBDNF and its hyperdigested form (ΔHSDPAR) have been measured in different fractions by RP-UPLC analysis. In fractions 6 and 7, the correctly processed protein is heavily contaminated by its hyperdigested form. Fraction 10, containing most of the initial material, mainly corresponds to enzymatic digestion variant (EDG) forms VRR-BDNF and R-BDNF, as determined by mass analysis.

Samples	% Purity rhBDNF Total Area	% Area Hyper vs. % total Area rhBDNF
T_0_ Load	54.9	9.9
Fraction 1	N/Q ^1^	N/Q
Fraction 2	N/Q	N/Q
Fraction 3	N/Q	N/Q
Fraction 4	N/Q	N/Q
Fraction 5	79.6	12.2
Fraction 6	61.7	48.2
Fraction 7	70.2	35.4
Fraction 8	43.5	126.1
Fraction 9	N/Q	N/Q
Fraction 10	N/Q	N/Q

^1^ Not quantifiable.

**Table 3:** Example of rhBDNF–VSAR variant analysis after SP column. SP fractions have been quantified by RP-UPLC for the presence of rhBDNF and its hyperdigested variant. Fraction 5, corresponding to the main peak, contains the highest quantity of mature rhBDNF, with the lowest amount of hyperdigested form.

Samples	% Purity rhBDNF Total Area	% Area Hyper vs. % Total Area rhBDNF
T_0_ Load	35.6	6.7
Fraction 1	41.5	7.1
Fraction 2	57.2	0.3
Fraction 3	51.6	2.0
Fraction 4	52.6	1.8
Fraction 5	64.9	0.9
Fraction 6	44.5	14.0
Fraction 7	37.8	42.2
Fraction 8	47.0	69.8

**Table 4:** Representative HIC fraction analysis by RP-UPLC. In Fraction 11, rhBDNF represents almost half of the eluted material. The other detected proteins are hypodigested variants, eluting in several distinct peaks, which are quantified but not formally identified by this method.

Samples	% Purity rhBDNF Total Area	% Area Hyper vs. % Total Area rhBDNF
T_0_ Load	84.6	0.2
Fraction 1	100.0	0.0
Fraction 2	97.5	0.1
Fraction 3	98.8	0.1
Fraction 4	99.1	0.0
Fraction 5	99.3	0.0
Fraction 6	99.4	0.0
Fraction 7	99.4	0.0
Fraction 8	100.0	0.0
Fraction 9	100.0	0.0
Fraction 10	98.5	0.3
Fraction 11	51.1	0.6

## Data Availability

The original contributions presented in this study are included in the article/Supplementary Materials, any further inquiries can be directed to the corresponding author.

## Supplementary Materials

The following supporting information can be downloaded at: https://www.mdpi.com/article/10.3390/ijms252413425/s1.

## Abbreviations

BDNF, brain-derived neurotrophic factor; NGF, nerve growth factor; NT-3, neurotrophin-3; NT-4,5, neurotrophin 4-5; OD, optical density; rh, recombinant human; ProBDNF, BDNF precursor; RP-UPLC, reversed-phase ultra performance liquid chromatography; LC–MS, liquid chromatography–mass spectrometry; Trk, tropomyosin-related kinase; NTs, neurotrophins; EDG, enzymatic digestion; EDTA, ethylenediaminetetraacetic acid; 4-MEP, 4-mercapto-ethyl-pyridine; SP, sulphopropyl; IPTG, isopropil-β-D-1-tiogalattopiranoside; HCIC, hydrophobic charge-induction chromatography; HIC, hydrophobic interaction chromatography; CEX, cation exchange chromatography; GMP, good manufacturing practice.
