# Supplementary material for: Efficient Production of Recombinant Human Brain-Derived Neurotrophic Factor in Escherichia coli Through the Engineering of Its Pro-Region
Source: Int J Mol Sci. 2024 Dec 14;25(24):13425. doi: 10.3390/ijms252413425 (PMC11678288; doi:10.3390/ijms252413425)
Supplement: Supplementary file 1 [file ijms-25-13425-s001.zip › ijms-3352758-supplementary.docx]

Article

Efficient production of recombinant human Brain Derived Neurotrophic Factor in *Escherichia coli* through the engineering of its Pro-region

Spaccapaniccia E.^1^, Cazzorla T. ^2^, Rossetti D. ^1^, De Simone L. ^1^, Antonangeli M.I. ^1^, Antonosante A. ^1^, Galli F. ^1^, Cattani F. ^1^, Maffei M. ^1^ and Martin F. ^1,*^

| **Citation:** To be added by editorial staff during production.  Academic Editor: Firstname Lastname  Received: date  Revised: date  Accepted: date  Published: date  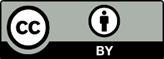  **Copyright:** © 2024 by the authors. Submitted for possible open access publication under the terms and conditions of the Creative Commons Attribution (CC BY) license (https://creativecommons.org/licenses/by/4.0/). |
| --- |

^1^ Dompé Farmaceutici S.p.A., Via Campo di Pile, Nucleo Industriale Pile, L’Aquila, 67100, Italy

^2^ Altadoc S.r.l., Via Della Stazione, 24, Celano, L’Aquila, 67043, Italy.

***** Correspondence: franck.martin@dompe.com

1. **Supplementary data**

**Supplementary Table 1.** LC Mass spectrometry results of fraction 10 of the SP HP chromatographic step for rhProBDNF WT form.

| **Sequence Name** | **MonoIsotopic**  **Mass (Da)** | **Theoretical**  **Mass (Da)** | **Fractional Abundance (%)** |
| --- | --- | --- | --- |
| R^111^-BDNF (EDG-V1) | 13652.8615 | 13652.8356 | 26.7% |
| V^109^R^110^R^111^-BDNF (EDG-V2) | 13908.0166 | 13908.0052 | 25.8% |
| Hyperdigested form (*Δ*HSDPAR) | 12833.4369 | 12833.4256 | 12.9% |
| rhBDNF | 13496.7553 | 13496.7345 | 8.7% |
| Other forms (Oxidized, Deamidated etc…) | -- | -- | 15% |
